# German adaptation of the Resources for Enhancing Alzheimer’s Caregiver Health II: study protocol of a single-centred, randomised controlled trial

**DOI:** 10.1186/1471-2318-14-21

**Published:** 2014-02-12

**Authors:** Stephanie Heinrich, Martin Berwig, Anke Simon, Jenny Jänichen, Nina Hallensleben, Witiko Nickel, Andreas Hinz, Elmar Brähler, Hermann-Josef Gertz

**Affiliations:** 1 Clinic and Policlinic for Psychiatry and Psychotherapy, Leipzig University, Semmelweisstr. 10, 04103 Leipzig, Germany; 2 Department of Applied Health Sciences, Business Department – Health Care Management, Baden-Wuerttemberg Cooperative State University Stuttgart, Tuebingerstr. 31-33, 70178 Stuttgart, Germany; 3 Department of Medical Psychology and Medical Sociology, Leipzig University, Philipp-Rosenthal-Strasse 55, 04103 Leipzig, Germany

**Keywords:** Dementia, REACH II, Intervention, Informal caregivers

## Abstract

**Background:**

Caring for a family member with dementia is extremely stressful, and contributes to psychiatric and physical illness among caregivers. Therefore, a comprehensive programme called Resources for Enhancing Alzheimer’s Caregiver Health II (REACH II) was developed in the United States to enhance the health of Alzheimer’s caregivers. REACH II causes a clear reduction of the stress and burdens faced by informal caregivers at home. The aim of this protocol is to adapt, apply, and evaluate this proven intervention programme in a German-speaking area for the first time. This newly adapted intervention is called Deutsche Adaption der Resources for Enhancing Alzheimer’s Caregiver Health (DeREACH).

**Methods:**

A total of 138 informal caregivers at home are recruited in a single-centred, randomised controlled trial. The intervention (DeREACH) consists of nine home visits and three telephone contacts over six months, all of which focus on safety, psychological well-being and self-care, social support, problem behaviour and preventive health-related behaviours. A complex intervention assessment on effectiveness will be adopted when the primary outcome – namely, the reduction of caregiver burden – and other secondary outcomes, including changes with regard to anxiety and depression, somatisation, health-related quality of life, and perceived social support, are measured at baseline, as well as immediately and three months after the intervention. The change from baseline to post-intervention assessment with regard to the primary outcome will be compared between treatment and control group using t-tests for independent samples.

**Discussion:**

It is anticipated that this study will show that DeREACH effectively reduces caregiver burden and therefore works under the conditions of a local German health-care system. If successful, this programme will provide an effective intervention programme in the German-speaking area to identify and develop the personal capabilities of informal caregivers to cope with the burdens of caring for people with dementia.

**Trial registration:**

NCT01690117

## Background

Caring for people with dementia is connected with enormous personal responsibilities and restrictions; therefore, family caregivers of people with dementia often feel more burdened and stressed than caregivers of older people with physical impairments [1]. Due to this high stress level, there is a higher risk of these informal caregivers becoming physically or mentally diseased [2]. For these caregivers, the conditions of experiencing stress and burden are complex [3], and the non-cognitive symptoms of dementia such as psychotic symptoms, depression, and challenging behaviour seem to play a significant role in this process [4].

American scientists created a wide-ranging programme regarding the development of effective interventions to support informal caregivers of people with dementia. In this programme, known as Resources for Enhancing Alzheimer’s Caregiver Health (REACH I) [5,6], the researchers tested, evaluated, and compared various single- and multi-component interventions. Based on these findings, Resources for Enhancing Alzheimer’s Caregiver Health II (REACH II), an intensive and individualised intervention with multiple components, was generated. REACH II causes a clear reduction of the stress and burdens faced by care-giving family members [6]. Nevertheless, despite its advantages and proven effectiveness, it has not been applied to German-speaking areas.

Hence, the planned study aims at the adaptation of the American REACH II intervention to the conditions of the German health-care system in the Deutsche Adaption der Resources for Enhancing Alzheimer’s Caregiver Health (DeREACH), as well as the evaluation of the DeREACH intervention (the official agreement of the developers of REACH II with this intention is on hand).

We assume that the DeREACH intervention as opposed to the standard supply of health-care services will reduce the burden of informal caregivers. Furthermore, positive effects on depression and somatisation as well as health-related quality of life and social support for care-giving family members can be expected.

## Methods

### Study design

The study has been designed as a non-blinded, randomised, and controlled trial. The intervention group receives the DeREACH intervention and the control group receives the standard supply of health-care services; both groups receive baseline, final, and follow-up assessments.

The design, conduct, and reporting of the DeREACH study will adhere to the Consolidation Standards of Reporting Trials (CONSORT) guidelines [7].

### Inclusion and exclusion criteria

We only include informal caregivers of people with dementia who live at home with their relatives and are residents of Leipzig. In addition, participants must be able to speak German fluently and a diagnosis of dementia should have been established by a physician for each care recipient.

### Recruitment of participants

The recruitment process is supported by the Geriatric and Gerontopsychiatric Network of Leipzig. This network was established to advance the collaboration of various health-care systems and to assure that people with dementia in particular receive constant attention. It involves memory clinics, home-care agencies, nursing homes, voluntary helpers, associations, and day-care facilitates. These participating facilities suggest eligible subjects, inviting them to attend the study. Furthermore, we address potential participants via articles and announcements in newspapers and via posters and flyers that are available in several health-care facilities.

### Randomisation

The participants are split into the study groups randomly. To obtain the same number of subjects in the intervention and the control group, we use a randomisation in blocks [8]. Moreover, we use a central randomisation via randomisation lists realised by an online procedure of the Medical Faculty of the Ludwig-Maximilians-University of Munich [9]. The outcome rater is not informed about the randomisation code of a participant before opening a sealed envelope with the code inside after completion of the baseline assessment.

### Blinding

The informal caregivers, their relatives with dementia, and the study staff cannot be blinded in matters related to the study group.

### Study procedure

A nurse from University Hospital Leipzig makes contact with our participants and arranges the first appointment. One or two outcome raters visit informal caregivers at home to conduct the baseline assessment. In addition, we undertake a dementia screening for diagnosing the degree of cognitive impairment [10]. This takes place during the first appointment at home with the diseased individual to confirm the diagnosis of dementia.

After completion of the baseline assessment, an envelope containing the subject’s randomisation code is opened to allocate the participant to the intervention group or control group.

Study staff will make another appointment for the first home visit with the informal caregivers allocated to the intervention group. Altogether, the informal caregivers in the intervention group receive nine home visits and three phone sessions. Every home visit is structured in its sequence and appointments will be scheduled visit by visit. Usually, the whole intervention lasts six months and a post-intervention assessment takes place immediately after completion. A follow-up assessment takes place nine months after the baseline assessment.

**Figure 1:**
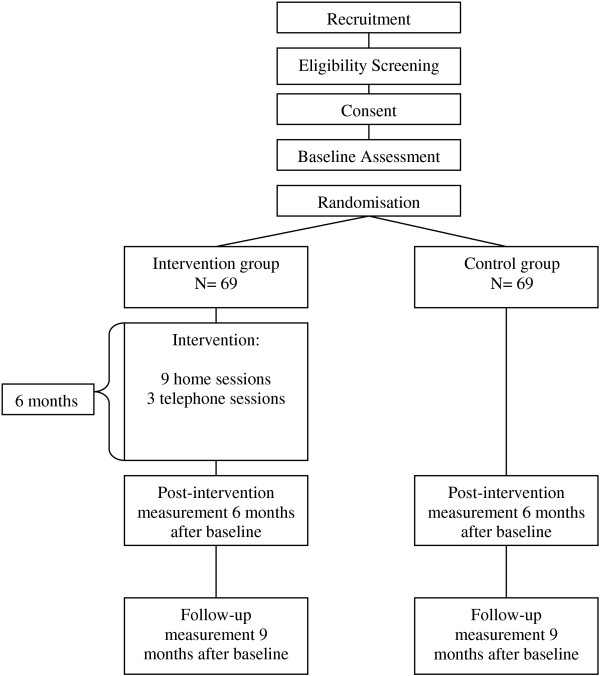
Study design.

The informal caregivers of the control group only receive the standard supply of health care services and the assessments outlined above. An overview of the study design and the assessment points is provided in Figure 1.

The intervention is executed by medical staff (for instance, nurses and occupational therapists). Prior to the intervention, these interventionists obtain training and a manual related to the intervention. Furthermore, they are attended and supported by research staff.

### Intervention

For this trial, the original REACH II intervention will be translated into the German language. Regional distinctions have to be adapted to meet conditions in Germany. Especially with regard to the areas of social support and self-care, it was necessary to include regional facilities and other offers of support. All 12 sessions of the DeREACH intervention are described in a study manual. Departing from the original REACH II intervention, we will train medical staff and not volunteers to perform the intervention. Another feature that required modification was the computer telephone integration system that the REACH II intervention provided to all participants, because the requirements are not available in the study area.

DeREACH is a multi-component intervention involving various strategies and techniques, such as psychoeducational information, problem-solving tasks, risk management, role playing, stress management, and social support. Five potential areas of risk in care giving receive particular attention in the intervention: safety, emotional well-being and self-care, social support, problem behaviour and preventive health behaviour. The intervention occurs over six months and includes 12 individual sessions (nine at home and three by telephone). Table 1 outlines a typical home session. A detailed description of the original REACH II intervention is available at the Epidemiology Data Center of the University of Pittsburgh [11].

**Table 1:** Exemplary structure of an intervention session

**Prior to a session, the interventionist reviews the risk worksheet**		
1)	Brief explanation of the focus of the session and how it will be structured	
2)	Explanation of the participation in social support groups	
3)	Interventionist checks and evaluates with the caregiver the strategies, material, and use of the handbook offered in previous sessions	
4)	Interventionist identifies and initiates a problem-solving approach focusing on the caregiver’s individual risk behaviour or problems	
5)	Interventionist provides training in different stress management techniques	
6)	Closure for each session:	
	a)	establishing date/time of next session
	b)	briefly reviewing problem areas
	c)	briefly reviewing strategies used
	d)	helping the caregiver implement strategies in daily routine
At the conclusion of the session, the interventionist completes a protocol of the session		

### Outcomes

Demographic information from the informal caregivers as well as from the people with dementia will be gathered via a questionnaire during the baseline assessment. In this trial, the outcome of primary interest is the effect on the burden of care-giving. Caregiver burden is perceived as a multi-dimensional response to stressors associated with care giving. We use a translated version of the Zarit Burden Inventory (ZBI [12]). The ZBI is a widely used 22-item assessment tool for measuring the caregiver’s perceived burden [13,14]. The data will be gathered at baseline, after intervention completion (six months), and at the follow-up nine months after baseline. Secondary outcomes are changes in the depression [15], somatisation [16], health-related quality of life [17] and social support of the family caregivers [18]. In addition, with regard to the relative with dementia, two further secondary outcomes will be collected: the effects on health-care service utilisation and problem behaviour [19] (see Table 2).

**Table 2:** Measurements

**Point of measurement**	**Measures**
*Baseline assessment:*	
- caregiver sociodemographics	- sociodemographic data questionnaire
*Baseline assessment with regard to people with dementia:*	
- sociodemographic information	- sociodemographic data questionnaire
- degree of cognitive impairment	- SIDAM score
*Primary outcome:*	
- caregiver burden	- ZBI
*Secondary outcomes:*	
- depression	- PHQ-4
- somatisation	- PHQ-15
- health-related quality of life	- SF-12
- social support	- ESSI
*Secondary outcomes with regard to people with dementia:*	
- health-care service utilisation	- 1 question
- problem behaviour	- RMBC

**Note.** SIDAM = Structured Interview for Diagnosing Alzheimer-Type Dementia, Multi-Stroke Dementia, and Dementias of other Aetiology according to DSM-III-R and ICD-10); ZBI = Zarit Burden Inventory; PHQ-4 = Patient Health Questionnaire - 4 items; PHQ-15 = Patient Health Questionnaire -15 items; SF-12 = Short-Form Health Survey -12 items; ESSI = ENRICHD Social Support Inventory; RMBC = Revised Memory and Behaviour Checklist.

### Sample size calculation

The calculation of sample size was based on an updated meta-analysis determining the effectiveness of interventions for family caregivers of older adults [20]. In that study, a moderate mean effect size of 0.65 (95% CI [0.46-0.84,]) was determined for multi-component interventions on caregiver burden. Assuming this effect size as significant for DeREACH, which is a multi-component intervention, and using a t-test for two independent samples with a 0.05 two-sided significance level, a sample size of 51 patients per group was calculated to have 90% power for detecting a treatment effect of that size (calculated by G*Power [21]). Allowing for a drop-out rate of 35%, a total of 69 informal caregivers per group is planned.

### Data analysis

All analyses will be conducted using Statistical Packages for Social Sciences (SPSS) version 20.0 for Microsoft Windows. The change from baseline to post-intervention assessment (six months) on the ZBI (primary outcome) will be compared between treatment groups using t-tests for independent samples on all randomised informal caregivers (intention-to-treat population). In case of significant between-group differences at baseline, an analysis of the covariance will be used with the post-intervention score as a dependent variable and treatment group and baseline ZBI score will be used as additional covariates. Several SPSS methods with missing value imputation will be used, including expectation maximisation (EM) and multiple imputations. Secondary analyses of the primary outcome variable will be performed on informal caregivers treated per protocol (PP population). Secondary study outcomes will be subjected to an explorative analysis. Post-hoc analyses will be performed on the primary outcome variable in patient subgroups, defined for instance by age, sex, caregiver education, family relationship and cognitive ability of the care recipient at baseline. Secondary and post-hoc analyses will be explorative without multiplicity adjustment.

### Ethical considerations

The study design, study protocol, procedure and informed consent are approved by the ethics committees of the Leipzig University (ref. 217-12-02072012). Participation is voluntary and all participants will sign informed consent.

## Discussion

The study described in this paper is the first study in Germany to investigate the effectiveness of the adapted REACH programme. A significant reduction in burden for informal caregivers over the trial period is expected as a primary outcome. Feeling burdened is a substantial reason for somatic and mental illness among informal caregivers. The results of this study will provide further evidence to support the feasibility of the DeREACH programme for home-care agencies or other service providers in the German health-care system. The findings of the study will yield valuable information regarding how to manage more effectively problems in family care giving for individuals with dementia. In addition, the findings of this study will provide information for others to implement the DeREACH programme in different areas and different cities and communities.

### Trial status

Recruitment into the study started on 1 October 2012.

## Competing interests

The authors declare that they have no competing interests.

## Authors’ contributions

All authors contributed to the design and development of the study protocol in their areas of expertise. EB and HJG are the project coordinators. SH and MB are responsible for the conduct of the general study. WN planned the statistical analysis and AH is the biometric counsellor. JJ and NH participate in study procedure. All authors were responsible for the drafting for this paper and approved the final manuscript.

## Pre-publication history

The pre-publication history for this paper can be accessed here:

http://www.biomedcentral.com/1471-2318/14/21/prepub
